# Association of multidimensional poverty and tuberculosis in India

**DOI:** 10.1186/s12889-021-12149-x

**Published:** 2021-11-11

**Authors:** Dimpal Pathak, Guru Vasishtha, Sanjay K. Mohanty

**Affiliations:** 1 Assam Medical College & Hospital Dibrugarh, Barbari, Assam India; 2 International Institute for Population Sciences, Govandi Station Road, Deonar, Mumbai, Maharashtra 400088 India; 3 Department of Development Studies, International Institute for Population Sciences, Mumbai, India

**Keywords:** Multidimensional poverty, Non-poor, Poverty, Tuberculosis, India

## Abstract

**Background:**

Reduction of multidimensional poverty and tuberculosis are priority development agenda worldwide. The SDGs aims to eradicate poverty in all forms (SDG 1.2) and to end tuberculosis (SDG 3.3.2) by 2030. While poverty is increasingly being measured across multiple domains, reduction of tuberculosis has been an integral part of public health programmes. Though literature suggests a higher prevalence of tuberculosis among the economically poor, no attempt has been made to understand the association between multidimensional poverty and tuberculosis in India. The objective of this paper is to examine the association of multidimensional poverty and tuberculosis in India.

**Methods:**

The unit data from the National Family Health Survey-4, conducted in 2015–16 covering 628,900 households and 2,869,043 individuals across 36 states and union territories of India was used in the analysis. The survey collected information on the self-reported tuberculosis infection of each member of a sample household at the time of the survey. Multidimensional poverty was measured in the domains of education, health, and standard of living, with a set of 10 indicators. The prevalence of tuberculosis was estimated among the multidimensional poor and non-poor populations across the states of India. A binary logistic regression model was used to understand the association of tuberculosis and multidimensional poverty.

**Results:**

Results suggest that about 29.3% population of India was multidimensional poor and that the multidimensional poverty index was 0.128. The prevalence of tuberculosis among the multidimensional poor was 480 (95% CI: 464–496) per 100,000 population compared to 250 (95% CI: 238–262) among the multidimensional non-poor. The prevalence of tuberculosis among the multidimensional poor was the highest in the state of Kerala (1590) and the lowest in the state of Himachal Pradesh (220). Our findings suggest a significantly higher prevalence of tuberculosis among the multidimensional poor compared to the multidimensional non-poor in most of the states in India. The odds of having tuberculosis among the multidimensional poor were 1.82 times higher (95% CI, 1.73–1.90) compared to the non-poor. Age, sex, smoking, crowded living conditions, caste, religion, and place of residence are significant socio-demographic risk factors of tuberculosis.

**Conclusion:**

The prevalence of tuberculosis is significantly higher among the multidimensional poor compared to the multidimensional non-poor in India.

## Introduction

Globally, about 10 million people were suffering from tuberculosis with estimated 1.4 million deaths in 2019 [1]. Tuberculosis is the 9th leading cause of death worldwide [1]. Among 11 countries in the WHO South-East Asia region, five, namely Bangladesh, India, Indonesia, Myanmar, and Thailand, account for 95% of the infected tuberculosis cases [2]. HIV, diabetes, viral hepatitis, and empyema are some of the disease-related risk factors of tuberculosis [3–5]. The WHO has placed a multi-sectoral strategy to end tuberculosis by 2030.

Tuberculosis is a contagious disease, requiring long-term treatment and care, that has social stigma associated with it. The prevalence of tuberculosis varies with the living conditions and the social and economic characteristics of the households. Tuberculosis has been more prevalent among the economically poor since time immemorial [6]. Studies suggest a strong and positive association of tuberculosis with poverty, illiteracy, unemployment, and catastrophic health spending [2, 7–10]. Lack of access to health care, deficient nutrition, and inadequate living conditions are some of the causes of the spread of tuberculosis. Besides, scarcity and inadequate quality of food, overcrowded habitation, and homelessness escalate tuberculosis transmission [8].

Traditionally, poverty has been estimated only in the economic domain (referred to as money-metric poverty). In recent literature, the practice has been to measure poverty across multiple domains, including health, education, economy, and environment. A growing number of studies have estimated multidimensional poverty both globally and nationally [11, 12]. In the recent years, the Oxford Poverty and Human Development Initiative (OPHI) has been providing global estimates of multidimensional poverty for more than 100 countries [12]. The Sustainable Development Goal (SDG) 1 aims to eradicate extreme poverty in all its forms everywhere while SDG-3 aims to end the TB epidemic by 2030 [13]. Eradication of multidimensional poverty is a key priority development agenda globally, nationally, and locally.

In accordance with SDG 1 and 3, reduction of both tuberculosis and multidimensional poverty accorded high priority in India. While the positive association of money-metric poverty with tuberculosis is well established [7, 10, 14], deprivations in multiple domains, such as education, nutrition, morbidity, sanitation, and hygiene, may increase the vulnerabilities to the tuberculosis infections. In 2019, India, with an estimate of 2.6 million tuberculosis infections accounted for one-fourth of the global infections [1]. Between 2005 and 06 and 2015–16, the overall prevalence of tuberculosis in the country decreased from 418 persons to 305 per 100,000 population [15]. India is also home to 364 million multidimensional poor population [16], though multidimensional poverty had declined from 54.7% in 2005–06 to 27.5% by 2015–16 [17]. While the prevalence of both tuberculosis and multidimensional poverty has been declining in the country, the inter-state variations in both these variables are large. To our knowledge, no attempt has been made so far to understand the association of multidimensional poverty with tuberculosis in India. Besides, prior studies in India have mostly been carried out on small unrepresentative samples and are limited geographical coverage. The aim of this paper is to provide the estimates of tuberculosis among multidimensional poor and non-poor and understand the association of tuberculosis and multidimensional poverty in India.

## Methods

### Data

Data from the fourth round of the National Family Health Survey (NFHS-4), 2015–16, India was used in the analysis. The NFHS-4 is a nation-wide, cross-sectional, and large-scale demographic health survey conducted under the stewardship of the Ministry of Health and Family Welfare (MoHFW), Government of India. It used multi-stage stratified systematic sampling to draw representative estimates at the national and subnational levels (state/district level). In the first stage, villages in the case of rural areas and census enumeration blocks in the case of urban areas were selected through the probability-proportional-to-size method. Households were selected through a systematic sampling scheme in the second stage of the sampling. The survey successfully collected information from 601,509 households (with a 98% response rate), covering 2,869,043 individuals throughout the country. The survey used three sets of schedules, namely, the household schedule, the woman schedule, and the man schedule. The household schedule provided information on household amenities (type of house, cooking fuel, drinking water, sanitation, electricity, assets ownership, etc) and basic demographic details for each member of the household (age, educational attainment, caste, religion and relationship to head of the household). The woman schedule was canvassed to women in the 15–49 age group in the domains of fertility, maternal health, child health, nutritional status, mortality, contraception, etc. Similarly, the man schedule collected information on health, marriage, contraception, etc. from men aged 15–54 years. The detailed methodology, sampling design, and survey instruments have been given in the report [15].

The NFHS-4 provides six types of separate data files, namely household, birth, kids, women, men, and person’s data file. The person’s data file contains all information at the individual level, which includes demographic, educational attainment and some health-related information for all members in a household. It may be mentioned that the household characteristics, such as electricity, type of house, cooking fuel and household assets, are the same for every member of a household, while the individual characteristics, such as age, sex, educational attainment, and some biomarkers, vary for each member of the household. The individual data file was used to estimate the extent of multidimensional poverty and tuberculosis.

Three specific questions related to tuberculosis were asked to the respondents in the household schedule. First, a screening question on ascertainment of tuberculosis was canvassed (Question No-23). It read as “*Does any usual resident of your household suffer from tuberculosis?”.* If anybody in the household reported tuberculosis, two subsequent questions (Q-24 and Q-24A) were asked that read as “*Who suffers from TB in the household?” and “Has the person received medical treatment for it?”*. Further, information was collected on the place of treatment (private/public facility). Tuberculosis was either self-reported or reported by any some other member of the household. Of the 8718 sample households that have at least one member with tuberculosis, 96.2% had only one member infected with tuberculosis. The unit data of NFHS-4 is publicly available and can be accessed by registering at www.dhsprogram.com/data/.

### Methodology

Descriptive statistics, multidimensional poverty indices, and logistic regression model were used in the analysis. Descriptive analyses were carried out to estimate the prevalence of tuberculosis across socio-economic and geographic characteristics. We used the Alkire and Foster (AF) method to estimate multidimensional poverty indices in the states of India. The AF method uses a dual cut-off methodology. First, the poor were identified in each weighted indicator based on pre-determined threshold/cut-off points of deprivation for each indicator (column three of Table 1). For instance, all household members were considered to be deprived in a particular indicator, say electricity if the household did not have electricity. In the second step, the aggregation of deprivation was made. Multidimensional poverty was defined at a threshold of 33% across composite deprivation score in line with global multidimensional poverty literature [18]. We used the estimates of both tuberculosis and multidimensional poverty at the individual level. This is logical as the data on tuberculosis was collected for all members of a household, while multidimensional poverty (similar to the poverty headcount ratio) was estimated at the population level (percentage of population below a threshold level). However, many of the indicators used for estimating multidimensional poverty were at the household level and remained the same for each member in the household. We also included two additional indicators, that is, number of households with at least one-member suffering from tuberculosis and percentage of households who are multidimensional poor.

**Table 1:** Dimensions and indicators, deprivation cut-off points, mean and weights used in estimating multidimensional poverty, India, 2015–16

Dimension	Indicator	Deprived if	Weight	Mean	SD
Education	1. Years of Schooling	No household member (aged 14+) has completed at least eight years of schooling.	1/6	12.31	0.329
	2. Current School attendance	Any school-age child in the household (up to grade 8) is not attending school.	1/6	6.87	0.253
Health	3. Under nutrition	Any household member is malnourished, as measured by the body mass index for adults (BMI < 18.5) and by the height-for-age z-score calculated based on World Health Organization standards for children under age 5	1/6	42.17	0.494
	4. Child mortality	Any child has died in the household within the five years prior to the survey.	1/6	3.75	0.19
Standard of living	5. Electricity	Household is not having access to electricity.	1/18	11.83	0.323
	6. Drinking water	Household is not having access to clean drinking water or having access to clean drinking water through a source that is located 30 min away or more by walking.	1/18	14.73	0.354
	7. Sanitation	Household is not having access to improved sanitation facilities or having access only to shared improved sanitation facilities.	1/18	52.02	0.5
	8. Cooking fuel	Household is using “dirty” cooking fuel (dung, wood or charcoal).	1/18	58.77	0.492
	9.Housing	The household has inadequate housing: the floor is made of natural materials or the roof or walls are made of rudimentary materials.	1/18	45.51	0.498
	10. Assets	The household does not own more than one of these assets: radio, TV, telephone, computer, animal cart, bicycle, motorbike, or refrigerator, and does not own a car or truck.	1/18	12.5	0.331

We used three broad dimensions - education, health, and standard of living - and a total of ten indicators in these three domains to define multidimensional poverty. Each indicator was directly or indirectly related to the SDGs. The inclusion criteria of the indicators and the deprivation cut off-points for measuring multidimensional poverty have been described elsewhere [18]. The deprivation cut-off point for the school attainment indicator was revised to completion of 8 years of schooling to suit the Indian context. The details of the dimensions, indicators, cut-off points, and weights are given in Table 1. Equal weights were assigned to each dimension, and within a dimension, equal weights were assigned to each indicator. The AF method provides three types of indices, that is, head count ratio, intensity of poverty, and multidimensional poverty index. The present study focuses on using multidimensional poverty since its prime objective was to estimates the prevalence of tuberculosis among multidimensional poor and non-poor. A brief description of the indices is given below.

Head count ratio (H): It is defined as the proportion of the multidimensionally poor to the total population and can be calculated as:

1 $$ H=\frac{q}{n} $$

Where *q* is the number of persons who are multidimensional poor and *n* is the total population. We have presented H as a percentage.

Intensity of poverty (A) is calculated as,

2 $$ A=\frac{\sum_1^qc}{q} $$

Where c is the deprivation score that the poor experienced. It is an average weighted count of deprivation experienced by the multidimensional poor.

Multidimensional poverty index (MPI) is the product of headcount ratio and intensity of poverty and calculated as:

3 $$ MPI=H\ast A $$

A chi-square test was carried out to understand the significant association between the prevalence of tuberculosis and the various characteristics considered in the study. A two sample proportions test was used to find out whether the difference between the prevalence of tuberculosis among the multidimensional poor and the non-poor was significant. We have also estimated Moran’s I statistic for tuberculosis and multidimensional poverty to see the extent of spatial clustering.

We used a binary logistic regression analysis to understand the risk factors associated with tuberculosis. Tuberculosis (the outcome variable) was categorized as 0 (absence of the disease) and 1 (presence of the disease). The most common mathematical equation used for the logistic regression modelling is as follows:

4 $$ logit(p)=\mathit{\ln}\left(\frac{p}{1-p}\right)={\beta}_0+{\beta}_1{X}_1+{\beta}_2{X}_2+\cdots +{\beta}_i{X}_i $$

Where p is the probability of having tuberculosis and *β*_*i*_ are the regression coefficients indicating the relative effect of changing a particular risk factor (the explanatory variable) on tuberculosis. The *X*_*i*_ are the independent (explanatory) variables considered in the study such as multidimensional poverty head count ratio, place of residence (rural or urban), religion, caste, smoking behaviour, overcrowding (defined as more than three persons living in one room), sex, and age. We did not included any such variable in the multivariate analysis that was used to estimate multidimensional poverty. We interpreted the results in terms of odds ratio (OR), with a 95% confidence interval and provided the robust standard error of the estimates. The confidence interval (CI) are based on robust standard error. All statistical analyses were performed in the STATA version 14 software package. All methods were carried out in accordance with relevant guidelines and regulations.

## Results

Table 1 presents the descriptive statistics of the variables used to estimate multidimensional poverty. Among 10 indicators, the largest deprivation in India was in cooking fuel, followed by sanitation, housing and nutrition. Deprivation was the lowest in child mortality, followed by school attendance.

**Fig. 1:**
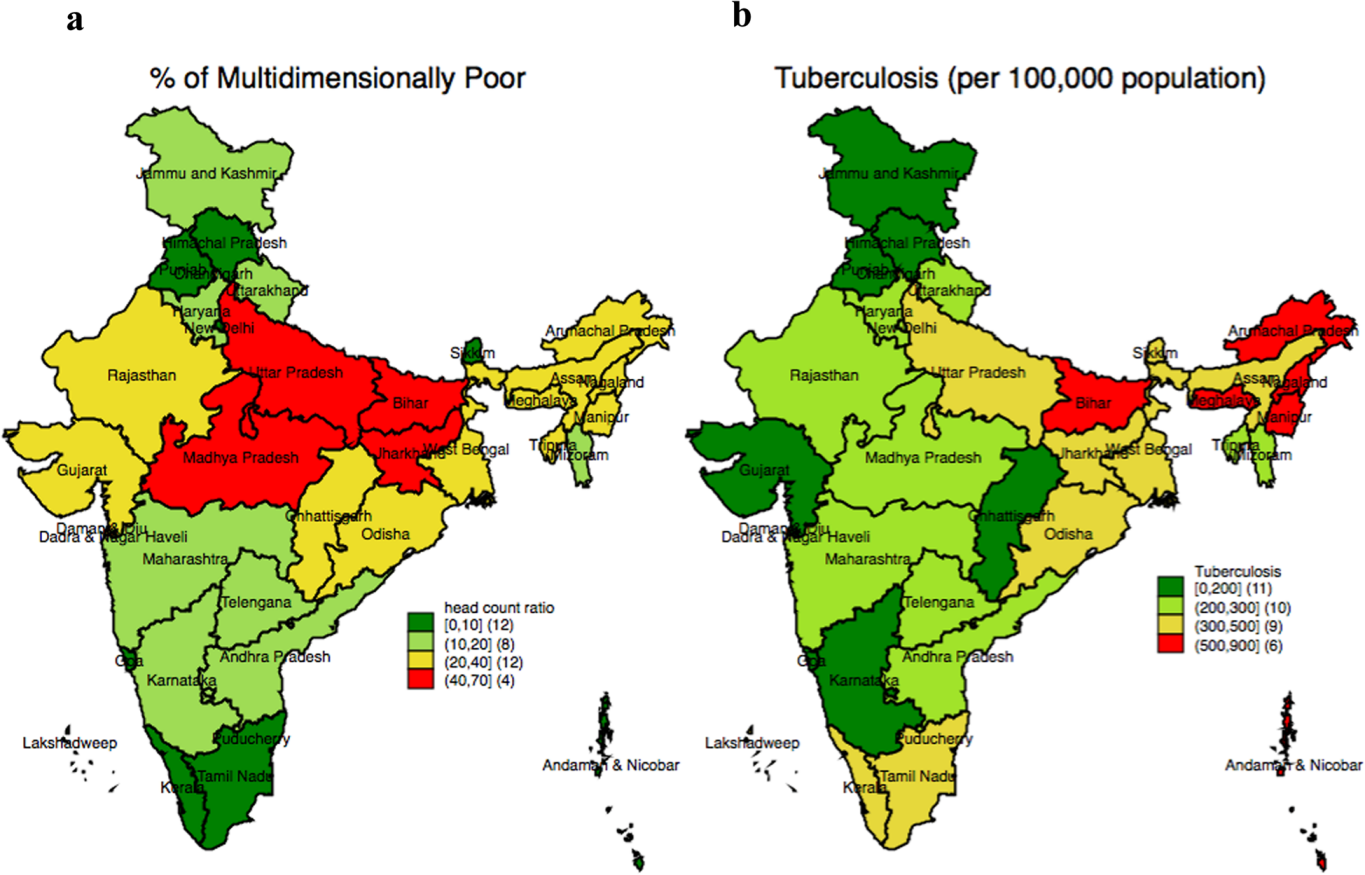
**a** Percentage of multidimensionally poor in the states of India, 2015–16; **b** Prevalence of tuberculosis (per 100,000 population) in the states of India, 2015–16

Figure 1a and b presents the state variations in percentage of the multidimensional poor and tuberculosis in India. The red shade in both of the maps shows the high prevalence of multidimensional poverty as well as tuberculosis in the states of India. There were four states, namely, Bihar, Uttar Pradesh, Jharkhand, and Madhya Pradesh, with over 40% point of multidimensional poor. A total of 12 states had multidimensional poverty between 20 and 40%, 8 states between 10 and 20%, and 12 states less than 10%. With regard to tuberculosis, we can see a strong regional pattern of clustering at a high level (> 500) in the north-eastern states of the country. A second category of states, mainly the states of southern India, had a higher prevalence of tuberculosis and the prevalence was relatively lower in the northern state of Punjab (150). In Bihar, the prevalence of both multidimensional poverty and tuberculosis was high.

**Fig. 2:**
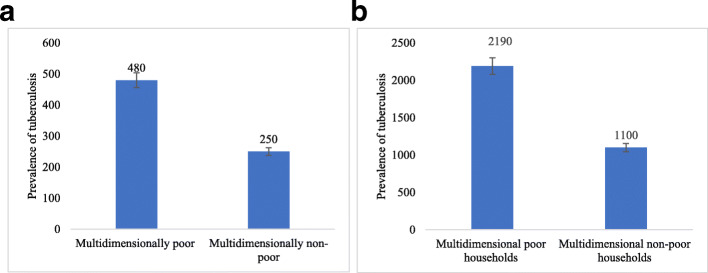
**a** Prevalence of tuberculosis by multidimensional poor and non-poor persons; **b** Prevalence of tuberculosis (at least one member in the household suffering from tuberculosis) by multidimensional poor and non-poor households in India, 2015–16

Table 2 shows the point estimates of percentage of multidimensional poor, intensity of poverty, the multidimensional poverty index, and the prevalence of tuberculosis (per 100,000 population) in the states of India. The estimates are provided for the entire sample population (individuals) and at the household level. In India, 29.3% population was multidimensional poor, and the MPI was estimated at 0.13. The extent of multidimensional poverty varied from 55.6% in Bihar to 1.0% in Kerala. The prevalence of tuberculosis in India was estimated at 310 per 100,000 population. The highest prevalence of tuberculosis was found in the state of Arunachal Pradesh (840 per 100,000 population), followed by Manipur (710 per 100,000 population) and Nagaland (650 per 100,000 population). The state variations in the prevalence of multidimensional poverty and tuberculosis were large. Figure 2 a shows the prevalence of tuberculosis among the multidimensional poor and multidimensional non-poor in India. The prevalence of tuberculosis among the multidimensional poor in India was 480 (per 100,000 population) [95% CI: 464–496] compared to 250 per (100,000 population) [95% CI: 238–262] among the non-poor. The prevalence of tuberculosis among the multidimensional poor households, with at least one member suffering from tuberculosis, was 2190 compared to 1100 among the multidimensional non-poor households (Fig. 2b). Such variations highlight the higher prevalence of tuberculosis among the multidimensional poor households in India. Moran’s I statistic of multidimensional poverty was 0.46 and that of tuberculosis was 0.38.

**Table 2:** Percentage of multidimensionally poor and intensity of poverty, multidimensional poverty index, and prevalence of tuberculosis (per 100,000 population) in the states of India, 2015–16

Sr. No	States	Percentage multidimensional Poor population (H)	Intensity of poverty (A%)	Multidimensional poverty Index	Prevalence of tuberculosis per 100,000 population (95% CI)	Prevalence of tuberculosis per 100,000 households (at least one member suffering from TB) (95% CI)	Percentage multidimensional Poor households (H %)
1	Kerala	1.03	37.74	0.003	370(366–374)	1400(1337–1464)	1.2
2	Lakshadweep	2.04	36.15	0.007	390(375–405)	2093(1800–2386)	0.9
3	Puducherry	3.68	36.26	0.013	140(134–146)	539(461–617)	4.4
4	Delhi	4.12	40.88	0.017	210(205–215)	951(877–1025)	3.6
5	Sikkim	5.53	37.87	0.021	470(463–477)	1831(1720–1942)	4.9
6	Chandigarh	5.37	42.68	0.023	90(80–100)	371(236–507)	4.3
7	Goa	6.49	36.53	0.024	70(64–76)	293(210–376)	5.5
8	Tamil Nadu	6.66	37.37	0.025	350(347–353)	1316(1275–1357)	8.7
9	Punjab	6.19	40.87	0.025	150(147–153)	725(686–765)	6.0
10	Andaman and Nicobar	7.17	38.69	0.028	610(600–620)	2425(2254–2596)	7.9
11	Daman and Diu	7.18	40.72	0.029	50(44–56)	189(120–257)	5.2
12	Himachal Pradesh	8.76	37.25	0.033	140(137–143)	609(560–658)	8.6
13	Haryana	11.25	42.25	0.048	230(227–233)	1138(1091–1186)	10
14	Mizoram	11.28	44.76	0.051	250(246–254)	1127(1069–1185)	9.9
15	Andhra Pradesh	14.37	40.84	0.059	300(296–304)	1187(1124–1250)	16.6
16	Telangana	16.33	40.49	0.066	300(295–305)	1170(1099–1241)	18.3
17	Karnataka	17.88	39.75	0.071	180(178–182)	757(724–791)	16.9
18	Jammu and Kashmir	17.35	41.37	0.072	160(158–162)	824(784–864)	14.5
19	Maharashtra	17.58	41.08	0.072	240(238–242)	1071(1034–1108)	16.2
20	Uttarakhand	18.29	41.42	0.076	250(247–253)	1119(1069–1169)	16.6
21	Tripura	20.59	42.6	0.088	250(244–256)	1011(923–1099)	21
22	Gujarat	22.15	42.3	0.094	180(178–182)	807(770–845)	19.8
23	Manipur	23.68	40.23	0.095	710(706–714)	3115(303–3199)	20.8
24	Nagaland	26.55	41.25	0.11	650(646–654)	2537(2456–2617)	23.1
25	Arunachal Pradesh	25.41	43.65	0.111	840(837–843)	3226(3150–3302)	23.6
26	West Bengal	27.01	41.59	0.112	350(346–354)	1417(1361–1472)	27
27	Rajasthan	32.94	44.97	0.148	210(208–212)	1067(1034–1099)	32
28	Dadra and Nagar Haveli	35.85	42.05	0.151	150(138–162)	714(530–898)	27.4
29	Meghalaya	34.94	44.38	0.155	550(545–555)	2675(2574–2776)	30.9
30	Chhattisgarh	37.85	41.15	0.156	160(158–162)	763(727–800)	38.5
31	Odisha	37.01	43.07	0.159	330(327–333)	1377(1338–1416)	36.4
32	Assam	38.22	44.22	0.169	310(307–313)	1382(1338–1425)	35.4
33	Madhya Pradesh	42.67	43.99	0.188	220(218–222)	1058(1031–1084)	40.9
34	Uttar Pradesh	44.42	44.48	0.198	330(329–331)	1748(1721–1775)	40.9
35	Jharkhand	49	44.32	0.217	320(317–323)	1544(1500–1588)	45.8
36	Bihar	55.58	46.85	0.26	640(638–642)	3282(3234–3330)	52.7
	**India**	**29.28**	43.68	**0.128**	**310(309–311)**	**1390(1381–1398)**	**27.8**

Table 3 presents the prevalence of tuberculosis per 100,000 population among the multidimensional poor and non-poor at the individual and household in the states of India. The variation in the prevalence of tuberculosis was higher among the multidimensional poor than among the non-poor individuals and households in most of the states of India. Among the multidimensional poor, the state of Kerala had the highest prevalence of tuberculosis (1590 per 100,000 population), followed by Delhi (1280 per 100,000 population) and Bihar (810 per 100,000 population). The prevalence of tuberculosis was lowest in Himachal Pradesh (220 per 100,000 population), followed by Jammu & Kashmir (230 per 100,000 population) and Chhattisgarh (260 per 100,000 population). Among the multidimensional non-poor, the prevalence of tuberculosis was the highest in Bihar (490 per 100,000 population), followed by Kerala (360 per 100,000 population) and Tamil Nadu (330 per 100,000 population). At household level, the prevalence of tuberculosis among multidimensional poor was highest in Delhi (6000 per 100,000 household) followed by Kerala (5162 per 100,000 households) and Bihar (2436 per 100,000 households). Similarly, the prevalence of tuberculosis among multidimensional nonpoor household was highest in Bihar (2436 per 100,000 households) followed by Uttar Pradesh (1382 per 100,000 households). The difference in the prevalence of tuberculosis among the multidimensional poor and non-poor was high and significant in most of the states of India. The pattern of the prevalence of tuberculosis by multidimensional poverty at individual and household levels was similar across the states of India.

**Table 3:** Prevalence of tuberculosis (per 100,000 population/households) among the multidimensional poor and non-poor in the states of India, 2015–2016

State	Number of TB cases per 100,000 Multidimensionally poor persons	Number of TB cases per 100,000 Multidimensionally non-poor persons	Difference (*P*-value)	Number of TB cases per 100,000 Multidimensionally poor household (at least one member in household suffering from TB)	Number of TB cases per 100,000 Multidimensionally non-poor household (at least one member in household suffering from TB)	Difference (*P*-value)
(1)	(2)	(3)	(4 = 2–3)	(5)	(6)	(7 = 5–6)
Andhra Pradesh	460	290	170 (0.014)	1582	1108	474 (0.0398)
Assam	490	210	280 (0.000)	2242	909	1333(0.000)
Bihar	810	490	320 (0.000)	4042	2436	1606(0.000)
Chhattisgarh	260	110	150 (0.000)	1148	523	625(0.000)
Gujarat	280	160	120 (0.002)	1332	678	654((< 0.0001)
Haryana	440	210	230 (0.000)	2277	1012	1265(0.000)
Himachal Pradesh	220	140	80 (0.308)	883	583	300(0.368)
Jammu and Kashmir	230	150	80 (0.001)	1230	755	475(< 0.0001)
Jharkhand	440	240	200 (0.000)	2060	1108	952(0.000)
Karnataka	300	160	140 (0.000)	1271	653	618(0.000)
Kerala	1590	360	1230 (< 0.0001)	5162	1354	3808(< 0.0001)
Madhya Pradesh	290	180	110 (0.000)	1388	829	559(0.000)
Maharashtra	310	230	80 (0.275)	1346	1018	328(0.4802)
Delhi	1280	170	1110 (0.000)	6022	760	5262(0.000)
Odisha	480	260	220 (0.000)	1937	1057	880(0.000)
Punjab	330	150	18 (0.035)	1542	674	868(0.0325)
Rajasthan	330	170	160 (0.000)	1599	816	783(0.000)
Tamil Nadu	720	330	390 (0.000)	2163	1235	928(< 0.0001)
Uttar Pradesh	440	280	160 (0.000)	2276	1382	894(0.0001)
Uttarakhand	360	240	120 (0.007)	1668	1010	658(0.003)
West Bengal	500	300	200 (0.005)	1937	1224	713(0.003)
India	480	250	230 (0.000)	2190	1100	1090(0.000)

*TB* Tuberculosis

Table 4 shows the prevalence of tuberculosis by selected sociodemographic factors among the multidimensional poor and non-poor (individuals) in India. The prevalence of the disease was higher among the multidimensional poor than non-poor across socio-demographic characteristics. Urban areas had a higher prevalence of tuberculosis (520 per 100,000 population) than the rural ones among both the multidimensional poor and the non-poor. The prevalence of tuberculosis (1300 per 100,000 population) was the highest among the elderly multidimensional poor. The prevalence increased with age among both the multidimensionally poor and the non-poor. The prevalence of tuberculosis among the multidimensionally poor males was 620 (per 100,000 population) compared to 350 (per 100,000 population) among the females. Prevalence tuberculosis was relatively more prevalent among smokers than non-smokers.

**Table 4:** Prevalence of tuberculosis (per 100,000 population) among the multidimensional poor and non-poor by background characteristics in India, 2015–16

Background and characteristics	Prevalence of TB (per 100,000 population) among multidimensional Poor	*P*-Value	Prevalence of TB (per 100,000 population) among multidimensional non-Poor	*P* -Value
**Age**				
< 15	70	< 0.0001	50	< 0.0001
15–44	460		180	
45–59	1150		460	
60+	1300		750	
**Sex**				
Male	620	< 0.0001	320	< 0.0001
Female	350		180	
**Crowding Condition**				
Over crowding	510	0.578	240	< 0.0001
No crowding	450		270	
**Smoking Behaviour**				
Not smoking	460	< 0.0001	210	< 0.0001
Smoking	490		320	
**Diabetes**				
Normal	450	< 0.0001	180	0.118
Diabetic	760		230	
**Place of Residence**				
Urban	520	< 0.0001	230	< 0.0001
Rural	470		270	
**Caste**				
SC	540	< 0.0001	290	< 0.0001
ST	450		310	
OBC	450		250	
Others	480		230	
**Religion**				
Hindu	470	< 0.0001	250	< 0.0001
Muslim	510		280	
Christian	660		360	
Others	480		210	
**Total**	480		250	

*TB* Tuberculosis

Table 5 gives the odds ratio of tuberculosis by multidimensional poverty and selected socio-demographic characteristics of respondents. The odds of suffering from tuberculosis was 1.8 times higher [95% CI: 1.75–1.91] among multidimensional poor compared to the multidimensional non-poor. The likelihood of having tuberculosis increased with age and was higher among the elderly. Smoking was found to be one of the significant predictors of tuberculosis. In comparison to Hindus, Muslims (OR 0.8, 95% CI: 1.21–1.38) had a lower risk of tuberculosis. Crowded living conditions were also to be a significant predictor of tuberculosis in India.

**Table 5:** Adjusted odds ratio for predicting tuberculosis by multidimensional poverty and selected risk factors in India, 2015–16

Background characteristics	Adjusted OR (95% CI)	Robust Standard Error	*P*- value
Multidimensional Poverty			
Multidimensional Non-Poor (Ref.)			
Multidimensional Poor	1.83***(1.74–1.91)	0.04	< 0.001
Age			
Less than 15 (Ref.)			
15–44	4.90***(4.47–5.36)	0.23	< 0.001
45–59	11.58***(10.55–12.73)	0.56	< 0.001
60+	16.04***(14.59–17.62)	0.77	< 0.001
Sex			
Female (Ref.)			
Male	0.56***(0.54–0.59)	0.01	< 0.001
Smoking Behaviour			
No Smoking (Ref.)			
Smoking	1.25***(1.2–1.30)	0.03	< 0.001
Crowding			
No crowding (Ref.)			
Over crowding	1.26***(1.20–1.32)	0.03	< 0.001
Place of Residence			
Urban (Ref.)			
Rural	1.11***(1.05–1.17)	0.03	< 0.001
Religion			
Hindu (Ref.)			
Muslim	1.29***(1.21–1.38)	0.04	< 0.001
Christian	1.99***(1.85–2.15)	0.07	< 0.001
Others	1.24***(1.13–1.37)	0.06	< 0.001
Caste			
Others (Ref.)			
SC	0.98 (0.91–1.05)	0.03	0.609
ST	0.84***(0.79–0.89)	0.03	< 0.001
OBC	0.70***(0.65–0.75)	0.03	< 0.001

Note: The odds ratio are adjusted for all variables in the table

**Fig. 3:**
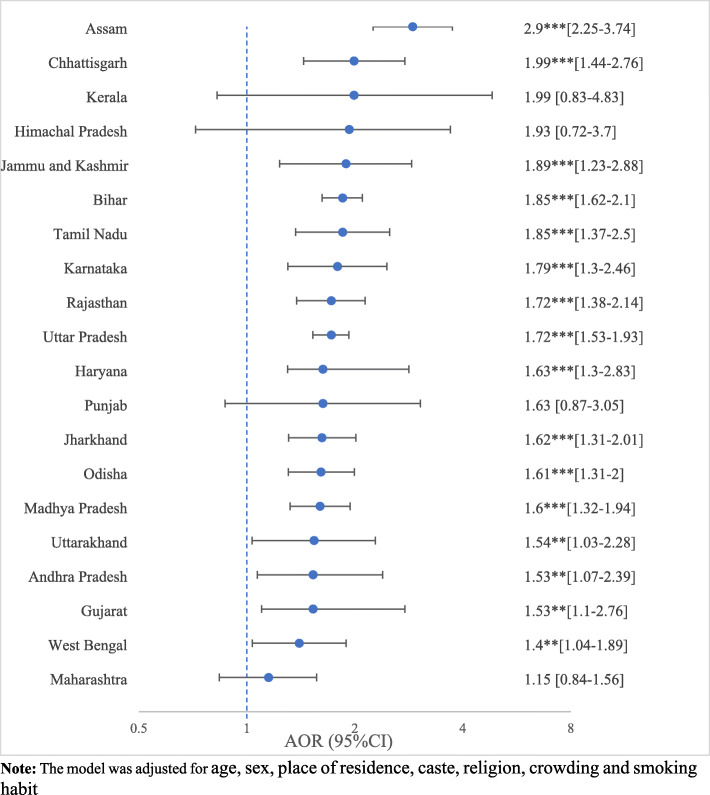
Adjusted odds ratio, with 95% confidence interval of multidimensional poverty gradient of tuberculosis, across the states of India, 2015–16. Note: The model was adjusted for age, sex, place of residence, caste, religion, crowding and smoking habit

Figure 3 presents the multidimensional poverty gradient of tuberculosis across states of India with help of adjusted odds ratio (OR). The OR are adjusted for age, sex, place of residence, caste, religion, crowding and smoking habit. Compared to the multidimensional non-poor, the multidimensional poor had significantly higher odds of having tuberculosis across the states of India. For instance, in Andhra Pradesh, the multidimensional poor were 53% (OR = 1.53; 95% CI: 1.07–2.39) more likely to suffer from tuberculosis compared to the multidimensional non-poor. Similarly, the multidimensional poor in Tamil Nadu had a more than 80% more likely to suffer from tuberculosis than the multidimensional non-poor. In the case of Assam, the chance of having tuberculosis was significantly higher among the multidimensional poor than the multidimensional non-poor. However, the states of Punjab, Kerala, Maharashtra, and Himachal Pradesh did not show significant variations in the prevalence of tuberculosis by multidimensional poverty.

## Discussion

Tuberculosis is a leading infectious disease and remains a major public health challenges in India. With 2.6 million tuberculosis infections and 364 million multidimensional poor, India has the largest number of tuberculosis patients and multidimensional poor worldwide. This is the first-ever study to have estimated the prevalence of tuberculosis among the multidimensional poor and non-poor across the states of India. We also examined the association of tuberculosis and multidimensional poverty. Our estimates of multidimensional poverty captures multiple domains of deprivation. The following are the salient findings of the study.

First, about one-third of the population in India was multidimensional poor, with large variations across the states of India. Multidimensional poverty was the highest in the state of Bihar (55.6%) and the lowest in Kerala (1.0%). These estimates are consistent with literature [19, 20]. The prevalence of tuberculosis was the highest in the north-eastern states of Arunachal Pradesh, Manipur, and Nagaland. The state variations observed in the study may be related to human paragonimiasis that occurs in Arunachal Pradesh, Manipur, and Nagaland, which is in turn associated with tuberculosis [21].

Second, the prevalence of tuberculosis among the multidimensional poor was estimated at 480 (per 100,000 population) compared to 250 (per 100,000 population) among the multidimensional-non-poor in India. The prevalence of tuberculosis was consistently higher among the multidimensional poor than the non-poor in most of the states and across most socio-demographic characteristics. The prevalence of tuberculosis among the multidimensional poor was higher in the economically and the educationally advanced states of Kerala and Delhi, as well as in the poorer states of Bihar. In the case of Kerala and Delhi, greater public awareness on tuberculosis and more availability of and accessibility to health services possibly resulted in a higher detection of the tuberculosis among the multidimensional poor. In the case of Bihar, the widespread prevalence of the disease may have been the probable reason for the high prevalence.

Third, the odds ratio from the logistic regression suggests that the multidimensional poor were significantly more likely to be reported with tuberculosis compared to the multidimensional non-poor. Smoking, overcrowding, religion, caste and age were all found to be significant predictors of tuberculosis. Many studies have established smoking as a significant risk factor that increases the risk of tuberculosis in individuals [22, 23]. Smoking increases the risk of progression from infection to disease or the risk of death from tuberculosis [24]. Literature suggests that people who live in overcrowded conditions come into contact with many other individuals, which can be considered a mechanism for the transmission of infectious diseases like tuberculosis [25, 26]. In terms of age, the highest tuberculosis prevalence occurs among the elderly. The multivariate analysis supports that the risk of susceptibility to tuberculosis increases with age.

Our finding as to the higher prevalence of tuberculosis among the multidimensional poor may be related to the fact that people, who are more likely to live and work in poorly ventilated and overcrowded conditions, do not avail quality treatment, which contributes to the spread of the tuberculosisbacteria [27]. Educational achievements are also an important factor in the reduction of tuberculosis [28, 29]. People with no formal education have a poorer knowledge of tuberculosis [30], and its treatment, which is, in turn, associated with an increase in transmission, treatment failure, and death. Awareness of diseases and infections and their spread can be considerably ramped up with increase in educational attainment [31].

The Revised National Tuberculosis Control Program (RNTCP) was launched in 1997 by adopting and formulating the internationally recommended DOTS strategy. In 2006, the WHO launched a new six-point “Stop TB” strategy to achieve the 2015 TB-related Millennium Development Goals. To eliminate tuberculosis in India by 2015, RNTCP formulated a National Strategic Plan for Tuberculosis 2017–25 with four strategic pillars Detect-Treat-Prevent-Build (DTPB). Despite the government giving free medicines and giving priority to reducing the disease, the prevalence of tuberculosis has remained high in India. The cases of drug-resistant tuberculosis have also been increasing and are a cause for concern. Tuberculosis can be significantly controlled by make individuals more aware of all aspects of the tuberculosis disease and create a supportive environment for tuberculosis the patients.

The study has a few limitations. First, the study was based on self-reported tuberculosis gathered by NFHS-4. It is possible that in some cases the household respondent may have not revealed the true information. Therefore, the estimated prevalence of tuberculosis is likely to be lower than the actual prevalence in the country. Second, the study may also suffer from the shortcoming of biased reporting of tuberculosis by a respondent on behalf of other household members. Third, the analysis of the association between tuberculosis and multidimensional poverty was limited to the variables available in the data set. Information on the state of health infrastructure and the accessibility to and quality of health services was not available in the data set. Despite these limitations, the findings of the study have make a significant contribution to the literature on domain of tuberculosis and multidimensional poverty in India.

The study suggests integrating multiple deprivations in the RNCTP programme so as to effectively implement the “End TB strategy” and reduce the prevalence of the disease among the multidimensional poor. Besides, there is a need to improve social support and increase public awareness to eliminate the stigma attached to tuberculosis,. Comprehensive and long-term interventions are recommended to eliminate tuberculosis in the country.

## Data Availability

The unit level data is available online in the Demographic Health Survey (DHS) data repository and can be accessed upon request at www.dhsprogram.com/data/ (DHS, 2018).
